# The Effects of Diverse Exercise on Cognition and Mental Health of Children Aged 5–6 Years: A Controlled Trial

**DOI:** 10.3389/fpsyg.2021.759351

**Published:** 2021-12-08

**Authors:** Ningxin Jia, Xijin Zhang, Xu Wang, Xiaosheng Dong, Yanan Zhou, Meng Ding

**Affiliations:** ^1^College of Physical Education, Shandong Normal University, Jinan, China; ^2^Mengyin Experimental Middle School, Linyi, China; ^3^Quanxin Primary School, Jinan, China; ^4^School of Physical Education, Shandong University, Jinan, China

**Keywords:** diverse exercise, cognition, mental health, children, intelligence

## Abstract

The rate of learning and cognitive development is at its highest level in preschool-aged children, making this stage a critical period. Exercise has received increasing attention for its beneficial physical and mental health effects on the development of preschool children. This study investigated the effects of diverse exercise on the cognition of preschool children. Two classes were randomly selected from kindergarten classes of children aged 5–6 years, and designated as the experimental and control classes. Each class contained 20 children (10 boys and 10 girls) according to the kindergarten class system. The experimental class completed exercises according to the designed curriculum, while control class carried out exercises according to the regular teaching plan, for a study period of 12 weeks. The Wechsler Preschool and Primary Scale of Intelligence (WPPSI) and the Mental Health Questionnaire for Children were used to assess outcomes, both at the beginning and end of study. After 12 weeks, the experimental class has improved in the “Object Assembly,” “Block Design,” “Picture Completion,” and “Coding” (14.70 ± 2.14, *p* < 0.01; 14.54 ± 1.56, *p* < 0.01; 9.62 ± 2.06, *p* < 0.05; 15.92 ± 2.72, *p* < 0.05) in performance test, and showed improvements in the “Movement,” “Cognitive Ability,” “Sociality” and “Living Habits” (5.65 ± 0.59, *p* < 0.01; 11.20 ± 1.91, *p* < 0.05; 9.05 ± 1.72, *p* < 0.05; 7.10 ± 1.45, *p* < 0.05) in mental health outcomes. Diverse exercise has a significantly beneficial role in promoting the cognitive development of children aged 5–6 years, as well as a beneficial, albeit insignificant, role in their mental health.

## Introduction

The rate of learning and cognitive development is at its highest level in preschool-aged children, making this stage a critical period (Sarıkaya and Coşkun, 2015). Providing children with high-quality interventions during this educational period will positively impact their cognitive development (Clark and Kingsley, 2020).

Exercise has received increasing attention for its beneficial physical and mental health effects on the development of preschool children. Evidence supports that exercise exerts a positive impact on cognition through several mechanisms, including glucose delivery, angiogenesis and neurotransmitter levels (Alvarez-Bueno et al., 2017). Furthermore, exercise can stimulate cognitive development in children, especially those in preschool (Bidzan-Bluma and Lipowska, 2018; Norris et al., 2020). Skills and relationships learned during exercise have a continuous impact on other aspects of children’s learning (Bidzan-Bluma and Lipowska, 2018). In primary schools and kindergartens, introducing exercise as an effective complementary activity can play a positive role in children’s learning (Norris et al., 2020). According to Piaget’s cognitive development theory, motor activity is essential for operational intelligence, especially spatial thinking (Lawrence et al., 1957). The theory indicates that action is the source of perception and the basis of thinking, and children’s psychological development is the result of the subject adapting to the object through action (Piaget, 1952). Piaget emphasized that physical and motoric experiences are necessary for the child’s conceptual development of movement and speed, especially during the early stages of development (Piaget et al., 2013).

Children’s cognitive abilities may be influenced by cardiopulmonary function and sedentary activity (Riso et al., 2019). Some systematic reviews on simple aerobic exercise have found that more exercise and less sedentary behavior can have certain health benefits for the physical, cognitive, social and emotional development of preschool children (Carson et al., 2017; Kuzik et al., 2020). The reason may be that long-term exercise enhances the expression of nerve growth factor, thereby improving the executive control network function and the development of cognitive brain function (Donnelly et al., 2016).

A positive correlation between exercise and academic performance has been observed among children, presumably due to changes in cognition, including executive function, memory, and fluid intelligence (Tomporowski et al., 2015). Many studies have focused on the positive effects of exercise on the prefrontal cortex and the hippocampus. Increased activation in the prefrontal cortex, involved in cognitive control, has been observed after exercise interventions (Sanchez-Lopez et al., 2019; Sember et al., 2020). Individuals with better cognitive development in early childhood appeared to be better prepared for learning and had stronger academic ability throughout the educational process (Bryant et al., 2020), and children with higher cognitive ability showed stronger physical fitness (Latorre-Roman et al., 2020).

However, previous studies have been more focused on simple aerobic exercise and the physical development of children (Alesi et al., 2016; Bezerra Alves and Alves, 2019; Bryant et al., 2020; Cai et al., 2020; Madsen et al., 2020; Williams et al., 2020). To the best of our knowledge, no studies have examined the effects of diverse exercise and its potential effects on preschool children. Thus, it is worth studying whether diversity and interest in exercise should be taken into account when arranging exercise courses in order to meet the developmental needs of children and improve their cognitive development. Participation in diverse exercise may be beneficial to the development of cognitive flexibility and working memory in preschool children. Exercise at an early age exhibits more benefits than that at later ages; for instance, a positive childhood lifestyle may have a protective effect on brain health later in life (Sanchez-Lopez et al., 2019). Diverse exercise involves all parts of the body, and the trajectories, routes and nature of the movement of different parts are varied. Children must focus on observing changes in movements to perform the correct action, which could increase their ability to observe and to block out distractions. Several sources have suggested that diverse play-based exercise could be particularly suitable for preschool children and provide more benefits than a single exercise (Scalise et al., 2018). We speculate that diverse exercise will be beneficial to the cognitive development of preschool children if the exercise curriculum is well designed and able to achieve the desired effect. At the same time, the content of the kindergarten curriculum will be enriched, and the exercise interest and experience of preschool children will be enhanced. It is especially important to investigate the effects of an appropriate exercise intervention on preschool children to strengthen their cognitive development. Since an increasing number of experimental studies have reported the mental health benefits of participating in exercise among preschool children (Donnelly et al., 2016), whether diverse exercise can improve the mental health of preschool children also deserves to be studied.

In view of this, in the present study, experiments were designed to test the hypothesis that diverse exercise can exhibit beneficial effects on the cognition of preschool children and can play a secondary role in mental health. This study can provide an effective reference for improving the learning ability of children and promoting their overall physical and mental development.

## Materials and Methods

### Study Population

Participants were recruited from a kindergarten in Jinan, Shandong, China. Two classes, designated as the experimental and control classes, were randomly selected from kindergarten classes of children aged 5–6 years using computer-generated random numbers corresponding to all classes. Each class contained 20 children (10 boys and 10 girls) according to the kindergarten class system. After the pre-test of related outcomes, the experimental class completed exercises according to the designed exercise curriculum, and the control class carried out exercises according to the regular teaching plan. The instructor of the exercise in the experimental class was a physical education practitioner with formal normal education who did not participate in the test of related outcomes. The related outcomes of the experimental and control classes were assessed by the same testers, who conducted relevant learning and training in advance to ensure the accuracy of the test.

### Interventions

Children in the experimental class performed 60 min of moderate-intensity exercise three times per week for 12 weeks according to the designed teaching plan. The diverse exercise curriculum of the experimental class consisted of four parts. The first part was a preparation activity lasting about 10 min. In the second part, the children carried out gymnastic exercises for about 10 min for a complete set of movements with a beat of 4 × 8, that is, 4 sets of 8 repetitions. The third part was about 30 min of diverse exercise, including drilling, rolling, walking, running, climbing, jumping, throwing, and other movements, taking into account the development of children’s balance, coordination, flexibility, sensitivity, endurance, flexibility, and other qualities, while also focusing on the cultivation of children’s observation, logical thinking ability and team spirit. The fourth part was a relaxation activity lasting about 10 min. The total time was 60 min (Scalise et al., 2018).

Children in the control class were given regular simple aerobic exercise classes according to the kindergarten teaching plan. The duration of exercise in the control class was the same as in the experimental class, which was 60 min. The two classes attended exercise sessions at different times to avoid a time conflict.

Except for the different class times and content of the exercise program, all other activities in the kindergarten were the same between the experimental and control classes, ensuring the continuation of normal activities and rest. Parents were asked to ensure that the attendance rate was as high as possible and that no other physical training classes were conducted during the experiment.

### Outcomes

Tests were performed before and after the 12-week intervention. A cognitive test was administered to the children by professionals from Shandong Normal University with the assistance of kindergarten teachers. A mental health test in the form of a questionnaire was distributed to guardians, who completed it based on the actual situation of the children and submitted it the next day. All personnel received training on tests before they were administered. The results were recorded on paper and transferred to the computer within 24 h.

#### Cognitive Test

Wechsler Preschool and Primary Scale of Intelligence (WPPSI) was used, which highlights the cognitive aspects of children’s intellectual ability, including verbal and performance tests (Wechsler, 2012). The verbal test included five parts: “Information,” “Vocabulary,” “Arithmetic,” “Similarities,” and “Comprehension,” which was used to measure the ability of verbal learning, conceptual formation and assimilation capacity of language, ability to analyze and summarize and abstract thinking related to language. The performance test included five parts: “Zoo Locations,” “Picture Completion,” “Object Assembly,” “Coding,” and “Block Design.” which was used to measure fluid intelligence, spatial perception, visual organization and reasoning ability (Wechsler, 2012). Because our participants were Chinese children, we used the official Chinese translation of WPPSI and then carried out the test (Lin and Zhang, 2012). Internal consistency reliability for WPPSI was calculated using a split-half analysis with normative sample data. Reliability coefficients for the composite scales ranged from 0.88 to 0.97 (>0.7) (Grizzle, 2011). Factor analysis studies have shown that the series of scales of WPPSI have high construct validity. The correlation coefficient of the total table score was 0.89 (>0.7) (Wechsler, 2012).

#### Mental Health Test

Subjects were tested with the Mental Health Questionnaire for Children Aged 5–6 years, which is from the book *Mental Health Measurement* compiled by Song (2005). The test included five parts: “Movement,” “Cognitive Ability,” “Emotion and Will,” “Sociality,” and “Living Habits” with a total of 38 question items. The reliability of internal consistency was evaluated with Cronbach’s alpha coefficients. In this study, Cronbach’s alpha was 0.74 (>0.7). Bartlett’s test of sphericity and the Kaiser–Meyer–Olkin (KMO) measure were used to test construct validity. In this study, the KMO measure was 0.75 (>0.7). Reliability and validity were found to be acceptable.

#### Data Statistics

The results were statistically analyzed using the SPSS 22.0 software. Sample size was estimated using preliminary arithmetic items in the WPPSI verbal test by Cohen’s d method (Cohen, 1992): the means of data from different groups (11.72 ± 1.04 and 13.31 ± 1.19, *n* = 6 per group) were divided by pooled standard deviation to calculate the standardized effect size (Cohen’s d value = 1.423), the largest of which was then compared with Cohen’s d power table to determine minimum group size (*n* = 17) with sufficient statistical significance (5%) and power (90%). A paired *t*-test was used for intra-group control, and a two-way analysis of variance (ANOVA) was used with the factors “group” and “time” to test for interaction effects. *P* < 0.05 was considered statistically significant.

## Results

### Basic Characteristics of Subjects

As shown in Table 1, there were 20 students in each class, with a male-to-female ratio of 1:1. The average height in the experimental and control classes was 120.90 ± 5.74 cm and 120.80 ± 6.56 cm, respectively. The average weight in the experimental and control classes was 23.15 ± 4.78 kg and 24.70 ± 6.49 kg, respectively. The average age in the experimental and control classes was 72.60 ± 3.65 months and 73.08 ± 2.96 months, respectively. All of the above characteristics were statistically the same between the two classes and met experimental requirements.

**TABLE 1:** Characteristics of subjects.

Parameters	Experimental (*n* = 20)	Control (*n* = 20)
Gender (male/female)	10/10	10/10
Height (cm)	120.9 ± 5.74	120.8 ± 6.56
Weight (kg)	23.15 ± 4.78	24.70 ± 6.49
BMI (kg/m^2^)	15.64 ± 0.97	16.68 ± 2.02
Age (months)	72.60 ± 3.65	73.08 ± 2.96

*The data are presented as mean ± SD.*

### Evaluation Outcomes of Wechsler Preschool and Primary Scale of Intelligence

#### Verbal Test

As shown in Table 2, in the experimental class, there were no significant changes in the scores on any of the verbal test items except for the “Similarities” item, which showed a higher post-test score. In the control class, there were no significant changes between the pre- and post-test scores on any of the verbal tests.

**TABLE 2:** Scores of verbal test items of Wechsler Intelligence Scale in experimental class and control class before and after the experiment.

Verbal test	Experimental (*n* = 20)			Control (*n* = 20)			Time × group (p)
	Pre-test	Post-test	Intra-group control (p)	Pre-test	Post-test	Intra-group control (p)	
Information	9.77 ± 2.42	9.31 ± 1.55	0.436	10.25 ± 4.14	9.92 ± 3.20	0.738	0.860
Vocabulary	9.62 ± 1.92	9.07 ± 1.75	0.950	8.75 ± 1.82	8.08 ± 1.56	0.232	0.530
Arithmetic	13.15 ± 2.23	13.85 ± 2.54	0.201	11.50 ± 3.23	12.50 ± 3.53	0.097	0.933
Similarities	6.78 ± 2.55	9.69 ± 2.98	0.004*	6.33 ± 2.71	7.50 ± 2.28	0.142	0.267
Comprehension	10.08 ± 1.61	9.85 ± 2.51	0.721	9.33 ± 2.15	9.14 ± 2.98	0.643	0.365

*The data are presented as mean ± SD.*

*p < 0.05.

**FIGURE 1:**
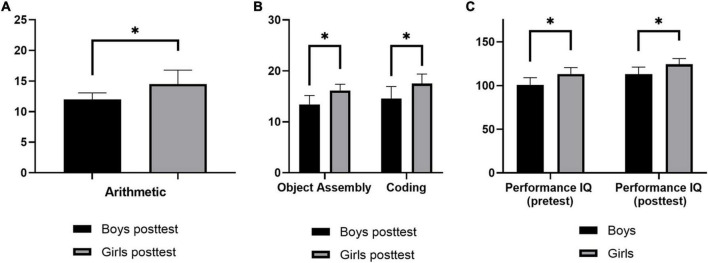
Difference between pre- and post-test measures according to sex. **(A)** Scores of “Arithmetic” in verbal test items of Wechsler Intelligence Scale in experimental class before the experiment. **(B)** Scores of “Object Assembly” and “Coding” in performance test items of Wechsler Intelligence Scale in experimental class after the experiment. **(C)** Scores of “Performance IQ” in IQ scores of Wechsler Intelligence Scale in experimental class before and after the experiment. *p < 0.05.

Table 3 shows that in the experimental class, there were no significant differences in the scores on any of the verbal test items between sexes, except for the “Arithmetic” item when comparing the pre-test scores between boys and girls. All significant differences measured according to sex are shown in Figure 1.

**TABLE 3:** Scores of verbal test items of Wechsler Intelligence Scale in experimental class according to sex before and after the experiment.

Verbal test	Boys (*n* = 20)			Girls (*n* = 20)			Inter-group control in pretest (P)	Inter-group control in posttest (P)
	Pre-test	Post-test	Intra-group control (P)	Pre-test	Post-test	Intra-group control (P)		
Information	10.29 ± 2.60	9.57 ± 1.29	0.558	9.16 ± 1.77	9.00 ± 1.63	0.880	0.430	0.531
Vocabulary	9.86 ± 2.10	9.14 ± 1.81	0.539	9.67 ± 1.49	9.00 ± 1.53	0.501	0.867	0.891
Arithmetic	12.00 ± 1.07	12.71 ± 1.91	0.439	14.50 ± 2.29	15.17 ± 2.34	0.659	0.037*	0.082
Similarities	6.43 ± 2.92	9.57 ± 2.61	0.073	7.17 ± 1.67	9.83 ± 3.13	0.124	0.625	0.883
Comprehension	10.29 ± 1.39	9.43 ± 2.87	0.523	9.83 ± 1.67	10.33 ± 1.60	0.640	0.634	0.541

*The data are presented as mean ± SD.*

*p < 0.05.

#### Performance Test

Table 4 shows the results of the performance test in the experimental class. When comparing pre- and post-test scores, all scores showed significant improvements, except for the “Zoo Locations” score. In the performance test in the control class, the “Zoo Locations” and “Picture Completion” scores did not show significant improvements. Similarly, the “Coding” score increased in the post-test, but the difference was not significant. The “Object Assembly” and “Block Design” scores showed significant improvements.

**TABLE 4:** Scores of performance test items of Wechsler Intelligence Scale in experimental class and control class before and after the experiment.

Performance test	Experimental (*n* = 20)			Control (*n* = 20)			Time × group (p)
	Pre-test	Post-test	Intra-group control (p)	Pre-test	Post-test	Intra-group control (p)	
Zoo locations	8.69 ± 2.10	8.84 ± 2.08	0.730	7.36 ± 1.93	8.50 ± 2.88	0.127	0.360
Picture completion	8.54 ± 1.05	9.62 ± 2.06	0.032*	7.42 ± 2.50	8.25 ± 2.99	0.107	0.391
Object assembly	12.92 ± 1.66	14.70 ± 2.14	0.002*	12.08 ± 5.43	11.08 ± 4.29	0.146	0.047*
Coding	14.31 ± 3.30	15.92 ± 2.72	0.030*	12.25 ± 4.58	13.67 ± 4.25	0.037*	0.396
Block design	10.27 ± 2.38	14.54 ± 1.56	0.000*	8.31 ± 3.93	10.58 ± 3.68	0.000*	0.041*

*The data are presented as mean ± SD.*

*p < 0.05.

As shown in Table 5, the post-test scores on “Object Assembly” and “Coding” were significantly improved when comparing boys and girls in the experimental class. The “Object Assembly” score showed significant improvements among girls in the post-test. Both boys and girls showed significant improvements in the “Block Design” item in the post-test. All significant differences measured according to sex are shown in Figure 1.

**TABLE 5:** Scores of performance test items of Wechsler Intelligence Scale in experimental class according to sex before and after the experiment.

Performance test	Boys (*n* = 20)			Girls (*n* = 20)			Inter-group control in pretest (p)	Inter-group control in posttest (p)
	Pre-test	Post-test	Intra-group control (p)	Pre-test	Post-test	Intra-group control (p)		
Zoo locations	8.00 ± 1.51	8.43 ± 2.50	0.726	9.50 ± 2.22	9.33 ± 0.94	0.880	0.212	0.457
Picture completion	8.57 ± 0.90	9.00 ± 1.69	0.594	8.50 ± 1.12	10.33 ± 2.05	0.110	0.909	0.263
Object assembly	12.29 ± 1.83	13.43 ± 1.76	0.292	13.67 ± 0.75	16.17 ± 1.21	0.003*	0.140	0.013*
Coding	12.86 ± 3.00	14.57 ± 2.38	0.294	16.00 ± 2.45	17.50 ± 1.89	0.304	0.086	0.047*
Block design	8.86 ± 2.17	13.86 ± 1.25	0.000*	11.33 ± 1.60	15.33 ± 1.37	0.002*	0.057	0.089

*The data are presented as mean ± SD.*

*p < 0.05.

### Evaluation Outcomes of Mental Health Outcomes

As shown in Table 6, when comparing the results of pre- and post-tests, the experimental class showed improvements in “Movement,” “Cognitive Ability,” “Emotion and Will,” “Sociality,” and “Living Habits” aspects to varying degrees, with significant differences in all outcomes except for “Emotion and Will.” On the other hand, there were no significant changes in the outcomes of the control class. There was no significant difference between the experimental and control classes in any of the outcomes except for the post-test “Movement” score.

**TABLE 6:** Mental health outcomes of experimental class and control class before and after experiment.

Mental health test	Experimental (*n* = 20)			Control (*n* = 20)			Time × group (p)
	Pre-test	Post-test	Intra-group control (p)	Pre-test	Post-test	Intra-group control (P)	
Movement	4.90 ± 0.97	5.65 ± 0.59	0.007*	5.24 ± 0.94	5.07 ± 1.14	0.382	0.019*
Cognitive ability	10.20 ± 2.07	11.20 ± 1.91	0.019*	10.76 ± 2.34	11.10 ± 1.79	0.406	0.219
Emotion and will	1.75 ± 0.44	1.80 ± 0.41	0.666	1.71 ± 0.46	1.52 ± 0.51	0.104	0.005*
Sociality	8.20 ± 1.66	9.05 ± 1.72	0.039*	9.04 ± 1.16	9.11 ± 1.64	0.890	0.066
Living habits	6.40 ± 1.96	7.10 ± 1.45	0.031*	6.48 ± 1.89	7.10 ± 1.30	0.148	0.460

*The data are presented as mean ± SD.*

*p < 0.05.

## Discussion

Several test items in the outcomes of WPPSI, such as “Picture Completion,” “Block Design,” “Coding” and “Object Assembly,” reflect the observation and perception abilities of preschool children. After 12 weeks of the exercise intervention, the experimental class significantly improved in “Object Assembly” and “Block Design” compared with the control class. These improvements demonstrate that the diverse exercise courses could be beneficial to the development of the observation and perception abilities of 5–6-year-old children.

The main reason for the beneficial effects of diverse exercise on children’s observation ability may be that, for the diverse exercise course, we designed several physical exercises that were beneficial to children’s development. Studies have focused on the positive effects of regular exercise on cognitive skills in children (Alesi et al., 2016). Cognitively engaging and coordinatively demanding activities are considered viable options to increase executive functions, potentially improving children’s cognitive skills and enhancing their attention and observation ability (Hsieh et al., 2017). Gymnastic exercises are based on the coherence and precision of movements and have a degree of standardization. Moreover, a complete set of exercises involves all parts of the body, and the trajectories, routes and nature of the movements of different body parts are varied. In addition, during the teaching process, we highlighted some key points and difficulties to explain and demonstrate separately, assigning certain practice tasks to the children with the aim of improving the pertinence and selectivity of their observation. The second reason for the observed beneficial effects of diverse exercise may be that sustained attention is correlated with enhanced memory performance (Johnson et al., 2019). Acute exercise upregulates neural activity in brain structures that play a key role in subserving episodic memory function, including the medial prefrontal cortex and medial temporal lobe (Kazuya et al., 2018). We designed jigsaw puzzles and included them in the game part of the diverse exercise course. For example, the children were allowed to carefully observe the jigsaw puzzle before playing the game, after which they divided them into pieces; then, they put the pieces together at the end of the activity. Recent studies have confirmed that engaging in an acute bout of exercise before a task requiring sustained attention can have additional effects on short-term memory function and improve task performance (Waters et al., 2020). Toward the end of the activity, the children performed various actions related to walking, running, jumping and throwing. After reaching the end of the activity, they fit the pieces together to form a meaningful whole action in the form of a relay race according to their memories. Children observed the jigsaw puzzle carefully before the game, requiring them to be selective and focus on the characteristics of the tasks due to limited time. Therefore, diverse exercise courses may strengthen the observation ability of 5–6-year-old children through purposeful and conscious cultivation of their observation ability. A previous randomized controlled trial performed a 4-week exercise program and concluded that daily brief coordinated exercise could improve attention in children (Harris et al., 2018). In our study, after a certain period of diverse exercise, the observation ability of the 5–6-year-old children was significantly improved.

The main explanation for the promoting effects of diverse exercise on children’s perception ability may be as follows: (1) Motion perception is the perception of objects moving in space, including the trajectory, speed and direction. After learning to imitate an action during gymnastic exercises, 5–6-year-old children may form a certain understanding of its direction and trajectory, which may also gradually strengthen during the practice. (2) Spatial perception refers to the reflection of the spatial characteristics of objects in the human brain, including orientation, shape, size, and depth. Short-term gymnastics training can generally promote children’s spatial working memory at the behavioral and neurophysiological levels (Aadland et al., 2019). Executive functions, including inhibition and spatial and auditory working memory, were significantly associated with agility, which was significantly linked to comprehensive physical ability *via* motor coordination (Aoyama and Imai-Matsumura, 2020). Diverse exercise, such as walking like a penguin on a balance beam, holding a sandbag on one’s head and forward rolling, requires children to perceive space, which benefits their spatial perception while developing their coordination and balance. This suggests that diverse exercise involving cognitive–motor interactions have potential benefits in the spatial cognitive ability of preschool children. (3) Time perception is the ability to manage the use of time when conducting tasks; the accuracy of time perception may rely on the executive control process related to the intentional allocation of attentional resources, including non-temporal stimulus properties (perception) (Matthews and Meck, 2016; Witowska et al., 2020). Diverse exercise includes tasks that preschool children must complete within a given time, so children could learn to adjust their performance of exercises in an attempt to complete the game within the allotted time. Through these exercises, children’s time perception was improved to a certain extent.

The scores improved in both genders after the intervention, but the post-test scores of the girls were higher. However, in the present study, this difference was less related to exercise and more likely due to the earlier development of girls. Girls peak in their physical growth earlier than boys, with a correspondingly earlier stabilization of brain metabolism (Vandekar et al., 2019). Some studies suggest that all language-related tracts develop earlier in girls than in boys, and girls have scored better than boys on reading tasks (Qiu et al., 2011; Lebel and Deoni, 2018; Kaczkurkin et al., 2019). The cognitive aspect of functioning yielded a higher correlation with motor functioning in female than in male children (Bala and Katić, 2009).

Maintaining a healthy lifestyle has been found to promote mental health in both neurotypical children and children with mental health problems (Sampasa-Kanyinga et al., 2020; Thomas et al., 2020). From the results of the mental health questionnaire, in terms of “movement” outcomes, after 12 weeks of the intervention, the experimental class showed greater improvement than the control class. This result indicates that the diverse exercise course was conducive to the development of movements in children between 5 and 6 years old and benefited their ability to learn basic movements. The main reason may be that the diverse exercise course included not only gymnastic exercises but also drilling, rolling, climbing, walking, running, jumping, throwing, and other various forms of exercise. Therefore, children aged 5–6 years could effectively exercise all parts of their body in the course of the exercise, and their balance, coordination, flexibility, sensitivity, endurance, and other aspects could be greatly improved. Previous reviews suggest an association between exercise and lower rates of depression and anxiety, or more positive self-perception and self-esteem, but the conclusions were limited due to the small sample size (Lubans et al., 2016; Rodriguez-Ayllon et al., 2019). While there is little evidence showing that exercise can significantly affect mental health in preschool children, a general trend has been observed suggesting that children who meet exercise recommendations have higher mental health scores (Rodriguez-Ayllon et al., 2019; Thomas et al., 2020). In terms of other outcomes, although the differences between the experimental and control classes did not reach statistical significance after the experiment, intra-group comparisons still revealed an improving trend in the experimental class with respect to “cognitive ability,” “sociality,” and “habits of life.” Although further study is needed to verify the benefits of diverse exercise on specific aspects of long-term conditions in children, increased participation in exercise should nevertheless be recommended as a potential means of improving long-term mental health (Dimitri et al., 2020).

However, it should be noted that while most studies report that 10–30 min of exercise exhibits beneficial effects on complex higher-level cognitive tasks (Byun et al., 2014), longer durations of exercise (>35 min) have been reported to negatively affect memory performance and decrease cognitive performance, likely due to the fatigue induced by prolonged exercise (Hacker et al., 2020). Furthermore, prolonged exercise is hypothesized to produce adverse effects due to an excessive increase in arousal (Crush and Loprinzi, 2017). Therefore, when designing a diverse exercise program, the exercise time should be reasonably controlled to ensure that the effects follow an optimal trend (Chang et al., 2012). In addition, exploratory analyses have indicated that the effect of acute exercise is temporally limited, with the intervention leading to accuracy gains only when tested directly after the intervention (Stein et al., 2017). Thus, long-term adherence needs to be taken into account in the teaching process, developing children’s exercise habits in order to ensure the enduring effect of diverse exercise. Although we have confidence in the reliability of test scores, which is typically assessed across time by administering the same test to the same individuals twice and then correlating the test and retest scores to produce a stability coefficient (Watkins and Smith, 2013; Brunner et al., 2017), the results should be interpreted with caution and further tested in future studies. Furthermore, the total physical activity of children was not assessed in the study, as children and their parents were instructed to reduce physical activity other than that in the intervention, which could be a potential limitation of the study.

## Conclusion

In conclusion, the results of this study indicate that diverse exercise has a beneficial role in the cognitive development of children aged 5–6 years. However, further studies with larger sample sizes and longer intervention times are needed to confirm its role in the mental health of children aged 5–6 years.

## Data Availability Statement

The original contributions presented in the study are included in the article/supplementary material, further inquiries can be directed to the corresponding author.

## Ethics Statement

The studies involving human participants were reviewed and approved by the Ethics Committee of College of Physical Education, Shandong Normal University. Written informed consent to participate in this study was provided by the participants’ legal guardian/next of kin. Written informed consent was obtained from the minor(s)’ legal guardian/next of kin for the publication of any potentially identifiable images or data included in this article.

## Author Contributions

MD planned the structure of the manuscript. NJ and XZ carried out the study and collected the important background information. NJ drafted the manuscript. XW, XD, and YZ assisted in data acquisition, data analysis, and statistical analysis. All authors have read and approved the final manuscript.

## Conflict of Interest

The authors declare that the research was conducted in the absence of any commercial or financial relationships that could be construed as a potential conflict of interest.

## Publisher’s Note

All claims expressed in this article are solely those of the authors and do not necessarily represent those of their affiliated organizations, or those of the publisher, the editors and the reviewers. Any product that may be evaluated in this article, or claim that may be made by its manufacturer, is not guaranteed or endorsed by the publisher.

## References

[B1] Aadland K. N. Ommundsen Y. Anderssen S. A. Bronnick K. S. Moe V. F. Resaland G. K. (2019). Effects of the active smarter kids (ASK) physical activity school-based intervention on executive functions: a cluster-randomized controlled trial. *Scand. J. Educ. Res.* 63 214–228. 10.1080/00313831.2017.1336477

[B2] Alesi M. Bianco A. Luppina G. Palma A. Pepi A. (2016). Improving Children’s coordinative skills and executive functions: the effects of a football exercise program. *Percept. Mot. Skills* 122 27–46. 10.1177/0031512515627527 27420304

[B3] Alvarez-Bueno C. Pesce C. Cavero-Redondo I. Sanchez-Lopez M. Alberto Martinez-Hortelano J. Martinez-Vizcaino V. (2017). The effect of physical activity interventions on children’s cognition and metacognition: a systematic review and meta-analysis. *J. Am. Acad. Child Adolesc. Psychiatry* 56 729–738. 10.1016/j.jaac.2017.06.012 28838577

[B4] Aoyama S. Imai-Matsumura K. (2020). Influences of executive functions on agility and comprehensive physical ability in kindergarteners. *Early Child Dev. Care* 2020, 1–10. 10.1080/03004430.2020.1773811

[B5] Bala G. Katić R. (2009). Sex differences in anthropometric characteristics, motor and cognitive functioning in preschool children at the time of school enrolment. *Coll. Antropol.* 33 1071–1078. 20102050

[B6] Bezerra Alves J. G. Alves G. V. (2019). Effects of physical activity on children’s growth. *J. Pediatr.* 95 S72–S78. 10.1016/j.jped.2018.11.003 30593790

[B7] Bidzan-Bluma I. Lipowska M. (2018). Physical activity and cognitive functioning of children: a systematic review. *Int. J. Environ. Res. Public Health* 15:800. 10.3390/ijerph15040800 29671803 PMC5923842

[B8] Brunner D. Abramovitch A. Etherton J. (2017). A yoga program for cognitive enhancement. *PLoS One* 12:e0182366. 10.1371/journal.pone.0182366 28783749 PMC5544241

[B9] Bryant L. M. Duncan R. J. Schmitt S. A. (2020). The cognitive benefits of participating in structured sports for preschoolers. *Early Educ. Dev.* 32 729–740. 10.1080/10409289.2020.1799619

[B10] Byun K. Hyodo K. Suwabe K. Ochi G. Sakairi Y. Kato M. (2014). Positive effect of acute mild exercise on executive function via arousal-related prefrontal activations: an fNIRS study. *Neuroimage* 98 336–345. 10.1016/j.neuroimage.2014.04.067 24799137

[B11] Cai K.-L. Wang J.-G. Liu Z.-M. Zhu L.-N. Xiong X. Klich S. (2020). Mini-basketball training program improves physical fitness and social communication in preschool children with autism spectrum disorders. *J. Hum. Kinet.* 73 267–278. 10.2478/hukin-2020-0007 32774558 PMC7386133

[B12] Carson V. Lee E.-Y. Hewitt L. Jennings C. Hunter S. Kuzik N. (2017). Systematic review of the relationships between physical activity and health indicators in the early years (0-4 years). *BMC Public Health* 17:854. 10.1186/s12889-017-4860-0 29219090 PMC5753397

[B13] Chang Y. K. Labban J. D. Gapin J. I. Etnier J. L. (2012). The effects of acute exercise on cognitive performance: a meta-analysis. *Brain Res.* 1453 87–101. 10.1016/j.brainres.2012.02.068 22480735

[B14] Clark G. F. Kingsley K. L. (2020). Occupational therapy practice guidelines for early childhood: birth-5 years. *Am. J. Occup. Ther.* 74 7403397010p1-7403397010p42. 10.5014/ajot.2020.743001 32365324

[B15] Cohen J. (1992). Statistical power analysis. *Curr. Dir. Psychol. Sci.* 1 98–101. 10.1111/1467-8721.ep10768783

[B16] Crush E. A. Loprinzi P. D. (2017). Dose-response effects of exercise duration and recovery on cognitive functioning. *Percept. Mot. Skills* 124 1164–1193. 10.1177/0031512517726920 28829227

[B17] Dimitri P. Joshi K. Jones N. (2020). Moving more: physical activity and its positive effects on long term conditions in children and young people. *Arch. Dis. Child.* 105 1035–1040. 10.1136/archdischild-2019-318017 32198161

[B18] Donnelly J. E. Hillman C. H. Castelli D. Etnier J. L. Lee S. Tomporowski P. (2016). Physical activity, fitness, cognitive function, and academic achievement in children: a systematic review. *Med. Sci. Sports Exerc.* 48 1197–1222. 10.1249/mss.0000000000000901 27182986 PMC4874515

[B19] Grizzle R. (2011). “Wechsler Intelligence Scale for children, fourth edition,” in *Encyclopedia of Child Behavior and Development*, eds Goldstein S. Naglieri J. A.. (Boston, MA: Springer US), 1553–1555.

[B20] Hacker S. Banzer W. Vogt L. Engeroff T. (2020). Acute effects of aerobic exercise on cognitive attention and memory performance: an investigation on duration-based dose-response relations and the impact of increased arousal levels. *J. Clin. Med.* 9:1380. 10.3390/jcm9051380 32397081 PMC7291087

[B21] Harris H. B. Cortina K. S. Templin T. Colabianchi N. Chen W. (2018). Impact of coordinated-bilateral physical activities on attention and concentration in school-aged children. *Biomed. Res. Int.* 2018 1–7. 10.1155/2018/2539748 29998131 PMC5994583

[B22] Hsieh S.-S. Lin C.-C. Chang Y.-K. Huang C.-J. Hung T.-M. (2017). Effects of childhood gymnastics program on spatial working memory. *Med. Sci. Sports Exerc.* 49 2537–2547. 10.1249/MSS.0000000000001399 28796655

[B23] Johnson L. Crawford L. Zou L. Loprinzi P. D. (2019). Experimental effects of acute exercise in attenuating memory interference: considerations by biological sex. *Medicina* 55:331. 10.3390/medicina55070331 31269780 PMC6680832

[B24] Kaczkurkin A. N. Raznahan A. Satterthwaite T. D. (2019). Sex differences in the developing brain: insights from multimodal neuroimaging. *Neuropsychopharmacology* 44 71–85. 10.1038/s41386-018-0111-z 29930385 PMC6235840

[B25] Kazuya S. Kyeongho B. Kazuki H. Zachariah M. R. Jared M. R. Akira M. (2018). Rapid stimulation of human dentate gyrus function with acute mild exercise. *Proc. Natl. Acad. Sci. U.S.A.* 115 10487–10492. 10.1073/pnas.1805668115 30249651 PMC6187140

[B26] Kuzik N. Naylor P.-J. Spence J. C. Carson V. (2020). Movement behaviours and physical, cognitive, and social-emotional development in preschool-aged children: cross-sectional associations using compositional analyses. *PLoS One* 15:e0237945. 10.1371/journal.pone.0237945 32810172 PMC7433874

[B27] Latorre-Roman P. A. Lloris-Ogallar E. Salas-Sanchez J. Garcia-Pinillos F. (2020). Association between executive function, intellectual maturity and physical fitness in preschoolchildren. *Revista Internacional De Medicina Y Ciencias De La Actividad Fisica Y Del Deporte* 20 471–485. 10.15366/rimcafd2020.79.006

[B28] Lawrence E. Piaget J. Inhelder B. Langdon F. Lunzer J. (1957). The Child’s conception of Space. *Br. J. Educ. Stud.* 5:187. 10.2307/3118882

[B29] Lebel C. Deoni S. (2018). The development of brain white matter microstructure. *Neuroimage* 182 207–218. 10.1016/j.neuroimage.2017.12.097 29305910 PMC6030512

[B30] Lin C. Zhang H. (2012). *The Revision of WPPSI-IV Chinese Version.* Beijing: Beijing Normal University.

[B31] Lubans D. Richards J. Hillman C. Faulkner G. Beauchamp M. Nilsson M. (2016). Physical activity for cognitive and mental health in youth: a systematic review of mechanisms. *Pediatrics* 138:e20161642. 10.1542/peds.2016-1642 27542849

[B32] Madsen M. Larsen M. N. Cyril R. Moller T. K. Madsen E. E. Orntoft C. (2020). Well-being, physical fitness, and health profile of 2,203 danish girls aged 10-12 in relation to leisure-time sports club activity-with special emphasis on the five most popular sports. *J. Strength Cond. Res.* 2020. 10.1519/JSC.0000000000003819 32991507

[B33] Matthews W. J. Meck W. H. (2016). Temporal cognition: connecting subjective time to perception. *Attent. Psychol. Bull.* 142 865–907. 10.1037/bul0000045 27196725

[B34] Norris E. van Steen T. Direito A. Stamatakis E. (2020). Physically active lessons in schools and their impact on physical activity, educational, health and cognition outcomes: a systematic review and meta-analysis. *Br. J. Sports Med.* 54 826–838. 10.1136/bjsports-2018-100502 31619381

[B35] Piaget J. (1952). *The Origins of Intelligence In Children.* New York, NY: International Universities Press.

[B36] Piaget J. Holloway G. E. T. Mackenzie M. J. (2013). *Child’s Conception of Movement and Speed.* London: Routledge.

[B37] Qiu D. Tan L. H. Siok W. T. Zhou K. Khong P. L. (2011). Lateralization of the arcuate fasciculus and its differential correlation with reading ability between young learners and experienced readers: a diffusion tensor tractography study in a Chinese cohort. *Hum. Brain Mapp.* 32 2054–2063. 10.1002/hbm.21168 21259386 PMC6870257

[B38] Riso E. M. Magi K. Vaiksaar S. Toplaan L. Jurimae J. (2019). Conceptual skills and verbal abilities were better in children aged six to seven years who were from more highly educated families and attended sports clubs. *Acta Paediatr.* 108 1624–1631. 10.1111/apa.14750 30740784

[B39] Rodriguez-Ayllon M. Cadenas-Sánchez C. Estévez-López F. Muñoz N. E. Mora-Gonzalez J. Migueles J. H. (2019). Role of physical activity and sedentary behavior in the mental health of preschoolers, children and adolescents: a systematic review and meta-analysis. *Sports Med.* 49 1383–1410. 10.1007/s40279-019-01099-5 30993594

[B40] Sampasa-Kanyinga H. Colman I. Goldfield G. S. Janssen I. Wang J. Podinic I. (2020). Combinations of physical activity, sedentary time, and sleep duration and their associations with depressive symptoms and other mental health problems in children and adolescents: a systematic review. *Int. J. Behav. Nutr. Phys. Act.* 17:72. 10.1186/s12966-020-00976-x 32503638 PMC7273653

[B41] Sanchez-Lopez M. Cavero-Redondo I. Alvarez-Bueno C. Ruiz-Hermosa A. Pozuelo-Carrascosa D. P. Diez-Fernandez A. (2019). Impact of a multicomponent physical activity intervention on cognitive performance: the MOVI-KIDS study. *Scand. J. Med. Sci. Sports* 29 766–775. 10.1111/sms.13383 30632640

[B42] Sarıkaya M. Coşkun E. (2015). A new approach in preschool education: social entrepreneurship education. *Proc. Soc. Behav. Sci.* 195 888–894. 10.1186/s12913-016-1423-5 27409075 PMC4943498

[B43] Scalise N. Daubert E. Ramani G. (2018). Narrowing the early mathematics gap: a play-based intervention to promote low-income preschoolers’ number skills. *J. Num. Cogn.* 3 559–581. 10.5964/jnc.v3i3.72 34553016 PMC8455118

[B44] Sember V. Jurak G. Kovac M. Morrison S. A. Starc G. (2020). Children’s physical activity, academic performance, and cognitive functioning: a systematic review and meta-analysis. *Front. Public Health* 8:307. 10.3389/fpubh.2020.00307 32760689 PMC7372103

[B45] Song Z. (2005). *Mental Health Measurement*, 2nd Edn. Guangzhou: JiNan University Press.

[B46] Stein M. Auerswald M. Ebersbach M. (2017). Relationships between motor and executive functions and the effect of an acute coordinative intervention on executive functions in kindergartners. *Front. Psychol.* 8:859. 10.3389/fpsyg.2017.00859 28611709 PMC5447760

[B47] Thomas M. M. Gugusheff J. Baldwin H. J. Gale J. Boylan S. Mihrshahi S. (2020). Healthy lifestyle behaviours are associated with children’s psychological health: a cross-sectional study. *Int. J. Environ. Res. Public Health* 17:7509. 10.3390/ijerph17207509 33076407 PMC7602583

[B48] Tomporowski P. D. McCullick B. Pendleton D. M. Pesce C. (2015). Exercise and children’s cognition: the role of exercise characteristics and a place for metacognition. *J. Sport Health Sci.* 4 47–55. 10.1016/j.jshs.2014.09.003

[B49] Vandekar S. N. Shou H. Satterthwaite T. D. Shinohara R. T. Merikangas A. K. Roalf D. R. (2019). Sex differences in estimated brain metabolism in relation to body growth through adolescence. *J. Cereb. Blood Flow Metab.* 39 524–535. 10.1177/0271678x17737692 29072856 PMC6421255

[B50] Waters A. Zou L. Jung M. Yu Q. Lin J. Liu S. (2020). Acute exercise and sustained attention on memory function. *Am. J. Health Behav.* 44 326–332. 10.5993/AJHB.44.3.5 32295680

[B51] Watkins M. W. Smith L. G. (2013). Long-term stability of the Wechsler Intelligence Scale for children–fourth Edition. *Psychol. Assess.* 25 477–483. 10.1037/a0031653 23397927

[B52] Wechsler D. (2012). *Wechsler Preschool and Primary Scale of Intelligence*, 4th Edn. Bloomington: Pearson.

[B53] Williams R. A. Cooper S. B. Dring K. J. Hatch L. Morris J. G. Sunderland C. (2020). Effect of football activity and physical fitness on information processing, inhibitory control and working memory in adolescents. *BMC Public Health* 20:1398. 10.1186/s12889-020-09484-w 32928161 PMC7488749

[B54] Witowska J. Zajenkowski M. Wittmann M. (2020). Integration of balanced time perspective and time perception: the role of executive control and neuroticism. *Pers. Individ. Differ.* 163:110061. 10.1016/j.paid.2020.110061

